# Synergistic Anticancer Effects of Lenvatinib Combined with N-butylidenephthalide in Human Colorectal Cancer Cells

**DOI:** 10.7150/ijms.122381

**Published:** 2026-03-04

**Authors:** Cheng-Chan Yu, Sung-Ying Huang, Shu-Fang Chang, Yu-Hung Kuo, Kuan-Fu Liao, Sheng-Chun Chiu

**Affiliations:** 1 Department of General Surgery, Taichung Tzu Chi Hospital, Buddhist Tzu Chi Medical Foundation, Taichung, Taiwan.; 2 Graduate Institute of Integrated Medicine, China Medical University, Taichung, Taiwan.; 3 Department of Ophthalmology, Hsinchu Mackay Memorial Hospital, Hsinchu, Taiwan.; 4 Department of Research, Taichung Tzu Chi Hospital, Buddhist Tzu Chi Medical Foundation, Taichung, Taiwan.; 5 Division of Hepato-gastroenterology, Department of Internal Medicine, Taichung Tzu Chi Hospital, Buddhist Tzu Chi Medical Foundation, Taichung, Taiwan.; 6 Department of Laboratory Medicine, Taichung Tzu Chi Hospital, Buddhist Tzu Chi Medical Foundation, Taichung, Taiwan.; 7 Division of Basic Medical Sciences, College of Nursing, Tzu Chi University, Hualien, Taiwan.

**Keywords:** colorectal cancer, lenvatinib, N-butylidenephthalide (BP), apoptosis, reactive oxygen species (ROS)

## Abstract

**Background:**

Colorectal cancer (CRC) is one of the most prevalent and deadly gastrointestinal malignancies worldwide. Lenvatinib, a multi-tyrosine kinase inhibitor, has shown limited clinical benefit as a monotherapy for CRC. Therefore, combining lenvatinib with N-butylidenephthalide (BP), a known anticancer and adjuvant agent, has been explored to enhance therapeutic outcomes. This study investigates the effects of lenvatinib and BP, individually and in combination, on HCT15 and HCT116 CRC cell lines.

**Methods:**

Cell proliferation was assessed by MTT assay. Cell cycle distribution and apoptosis were measured with PI staining and annexin V-FITC staining, respectively, analyzed by flow cytometry. Reactive oxygen species (ROS) levels were determined using the DCFDA assay. Mitochondrial membrane potential (MMP) was evaluated with the JC-10 assay. Oxidative DNA damage was quantified by measuring 8-hydroxy-2'-deoxyguanosine (8-OHdG) levels using an ELISA kit. Immunofluorescence staining was performed to evaluate γ-H2AX foci expression. Protein expression levels related to apoptosis and cell cycle regulation were analyzed by western blotting.

**Results:**

The combination of lenvatinib and BP exhibited synergistic cytotoxicity effect, promoting apoptosis by disrupting MMP in CRC cells. Additionally, this combination increased ROS accumulation, leading to oxidative DNA damage via 8-OHdG induction. Furthermore, the combination treatment induced G2/M phase cell cycle arrest by modulating the ATM-Chk2 signaling pathway.

**Conclusions:**

This study demonstrates that the combination of lenvatinib and BP represents a promising therapeutic strategy for CRC by enhancing apoptosis and cell cycle arrest through ROS-mediated DNA damage.

## Introduction

Colorectal cancer (CRC) is the most prevalent gastrointestinal malignancy and the third leading cause of cancer-related mortality worldwide 1. The global incidence continues to rise, with an estimated 3.2 million new cases anticipated by 2040 2. Current treatment options for CRC include surgical resection, radiotherapy, chemotherapy, and their combinations 3. However, over 30 % of CRC patients experience disease recurrence post-treatment, with a 5-year survival rate of approximately 13 % in advanced stages due to recurrence and metastasis 4. Therefore, there is an urgent need to develop effective therapeutic strategies to enhance chemotherapy efficacy against CRC.

Lenvatinib, a multi-targeted tyrosine kinase inhibitor, targets vascular endothelial growth factor receptors 1-3 (VEGFR1-3), fibroblast growth factor receptors 1-4 (FGFR1-4), platelet derived growth factor receptor-alpha (PDGFRα), c-kit, RET, and the mast/stem cell growth factor receptor (SCFR) to exert its therapeutic effects 5-7. While the U.S. FDA has approved the treatment of lenvatinib primarily for thyroid cancer, renal cell carcinoma, and hepatocellular carcinoma (HCC), its clinical benefits on other types of cancers are still being explored. Previous reports have indicated the anti-tumor efficacy of lenvatinib on human CRC cell lines *in vitro* and the suppression of angiogenesis in *KRAS*-mutant CRC xenograft mouse model* in vivo* 8, 9. In addition, a recent phase II study further highlighted the therapeutic potential of lenvatinib in patients with advanced CRC following the failure of standard chemotherapy 10. These studies provide experimental evidence for the potential clinical use of lenvatinib in the treatment of CRC. Besides, lenvatinib has shown synergistic effects when combined with paclitaxel in anaplastic thyroid cancer and with everolimus in metastatic renal cell carcinoma 11, 12. These findings underscore the value of developing synergistic combination therapies with lenvatinib for CRC treatment.

Recently, the naturally occurring compounds have been considered as complementary medicines cancer therapies due to their favorable safety profiles compared to conventional chemotherapy or targeted drugs 13-15. N-butylidenephthalide (BP; C_12_H_12_O_2_), one of the active components extracted from the root of *Angelica sinensis* (commonly known as *danggui* in traditional Chinese medicine), has been reported to exhibit anti-cancer effects against various types of cancer, including CRC 16-19. Specifically, the combination of BP with first-line chemotherapy drugs such as fluorouracil (5-FU) or cisplatin enhances sensitivity and ameliorates drug resistance in CRC and bladder cancer cells 20, 21. These findings suggest that BP is a potential candidate for exploring synergistic therapeutic approaches with minimal toxicity for CRC treatment.

Reactive oxygen species (ROS) play a pivotal role in cellular processes, contributing to both cell survival and death. Elevated ROS levels can induce DNA damage, triggering the DNA damage response (DDR), which regulates the cell cycle progression, activates DNA repair mechanisms, and may lead to cell death through the ATM/ATR-Chk1/2 signaling pathway 22, 23. Chemotherapeutic agents increase ROS levels, inducing DDR via nucleoside base oxidation, leading to genomic instability in cancer cells as a consequence of chemotherapy 24. Malignant cells have been reported to exhibit higher oxidative stress and elevated basal ROS levels compared to non-malignant cells, making them more susceptible to ROS-induced cell death. 25. Lenvatinib has been reported to induce cell death via ROS generation and ferroptosis induction in HCC cells 26, 27. Thus, novel combination regimens that amplify ROS production are potential candidates for CRC drug development.

This study aims to investigate the synergistic anticancer effects of BP in combination with lenvatinib in CRC treatment and elucidate the underlying mechanisms. Our findings demonstrate that this combination treatment inhibits cell proliferation, promotes apoptosis through MMP disruption, and induced G2/M phase cell cycle arrest via the ATM-Chk2 pathway, which was attributed to ROS-mediated DNA damage in human CRC cells.

## Material and Methods

### Chemicals and antibodies

N-butylidenephthalide (C_12_H_12_O_2_, 95 %) was purchased from Alfa Aesar (Ward Hill, NY, USA). Lenvatinib, sodium vanadate (Na_3_VO_4_), dimethyl sulfoxide (DMSO), [3-(4,5-dimethyl thiazol-2-yl)-2,5-diphenyl tetrazolium bromide] (MTT), DAPI, tween-20, methanol, and horseradish peroxidase-conjugated secondary antibodies were obtained from Sigma-Aldrich (St. Louis, MO, USA). Antibodies against p-H2AX (Ser139), p-Chk2 (Thr68), Chk2, cyclin B, p-cdc2 (Tyr15), cdc2, cdc25c, PARP, cleaved caspase-9, cleaved caspase-3, GAPDH, and β-actin were purchased from Cell Signaling Technology (Danvers, MA, USA). The Bradford protein assay kit was obtained from Bio-Rad (Hercules, CA, USA). PVDF membranes were purchased from Merck Millipore (Bedford, MA, USA). Western blot chemiluminescence reagents were purchased from Amersham Biosciences (Arlington Heights, IL, USA).

### Cell culture

Human colorectal cancer cell lines HCT15 and HCT116 were obtained from the Bioresource Collection and Research Center (BCRC, Hsinchu, Taiwan). The normal human colon epithelial cell line CCD 841 CoN was kindly provided by Prof. Chia-Che Chang (National Chung Hsing University, Taichung, Taiwan). Cells were cultured at 37 °C in a humidified atmosphere with 5 % CO_2_ in standard medium as recommended by the ATCC and BCRC. Culture medium, FBS, and supplements were purchased from Invitrogen (Carlsbad, CA, USA).

### MTT assay

Cell viability was evaluated using the MTT assay, as previously described 28. Fresh solutions of lenvatinib (10-80 μM) and BP (12.5-100 μg/ml) were prepared. Additionally, solutions containing 0.2 % DMSO (control), 20 or 40 μM lenvatinib, and 20, 40, or 80 μg/ml BP were freshly prepared in culture medium and applied to HCT15 and HCT116 cells. After 24 h of treatment, the drug-containing medium was removed, cells were washed with PBS, and culture medium containing 300 μg/ml MTT in culture medium was added for 4 h at 37 °C. The MTT medium was then removed, and 0.5 ml of DMSO was added to each well. Absorbance was measured at 570 nm using a multi-well plate reader Infinite 200 Pro Tecan^TM^ (Tecan, Mannedorf, Switzerland). The absorbance of DMSO-treated cells was set as 100 %.

### Western blot analysis

Western blot analysis was performed as previously described 28. CRC cells were lysed in a 6-cm plate using 100 µl of M-PER mammalian protein extraction reagent containing a protease inhibitor cocktail (Thermo Scientific, Rockford, IL, USA) and centrifuged at 13,000 × g at 4 °C for 10 min to collect the supernatant. Protein concentrations were determined using the Bradford assay. Electrophoresis was conducted on a NuPAGE Bis-Tris Electrophoresis System, loading 20 µg of reduced protein extract per lane. The resolved proteins were transferred to PVDF membranes, which were subsequently blocked with 5 % skim milk for 1 h at room temperature, and then probed with specific primary antibodies overnight at 4 °C. Following three washes with TBS/0.2 % Tween-20 at room temperature, the PVDF membrane was incubated with an appropriate secondary antibody labeled with horseradish peroxidase (Sigma Chemical, St. Louis, MO, USA) for 1 h at room temperature. All resolved protein bands were detected using Western Lightning™ Chemiluminescence Reagent Plus (Amersham Biosciences, Arlington Heights, IL, USA).

### Flow cytometric analysis

Cell cycle and apoptosis analyses were performed using flow cytometry, as previously described 28. CRC cells were treated with 80 μg/ml of BP, with or without 20 or 40 μM lenvatinib, and incubated for 24 h (for cell cycle analysis) or 48 h (for apoptosis analysis). For cell cycle analysis, cells were stained with a solution containing 20 μg/ml PI, 0.2 mg/ml RNase A, and 0.1 % Triton X-100 for 30 min in the dark to quantity total DNA content. Apoptosis was assessed using an annexin V-FITC detection kits (BD Biosciences, San Diego, CA, USA) according to the manufacturer's instructions. All analyses were conducted using a BD Accuri C6 flow cytometer (BD Biosciences, San Diego, CA, USA).

### DCFDA assay

ROS levels were measured using the 2',7'-dichlorofluorescein diacetate (DCFDA) assay, as previously described 29. Briefly, CRC cells were seeded in 6-cm dishes and treated with the indicated concentrations of BP or lenvatinib for 24 h. Cells were then incubated with 10 μM DCFDA at 37 °C for 30 min, washed with PBS, and analyzed using a BD Accuri C6 flow cytometer (BD Biosciences, San Diego, CA, USA).

### 8-hydroxy-2-deoxyguanosine (8-OHdG) Quantitation

CRC cells were seeded in 6-cm dishes and treated with 20 or 40 μM lenvatinib, with or without 80 μg/mL BP, for 48 h. DNA was extracted using the DNeasy Blood & Tissue Kit (Qiagen). Extracted DNA (2 mg/ml) was analyzed for 8-OHdG levels using the OxiSelect™ Oxidative DNA Damage ELISA kit (Cell BIOLABS, INC). Absorbance was measure at 450 nm using multi-well plate reader Infinite 200 Pro Tecan^TM^ (Tecan, Mannedorf, Switzerland).

### Immunofluorescence staining

Immunofluorescence was performed as previously described 30. CRC cells were seeded in 6-well plates and treated with 20 or 40 μM lenvatinib, with or without 80 μg/ml BP, for 24 h. Cells were washed with PBS, fixed with 4 % paraformaldehyde for 10 min at room temperature. Subsequently, the cells were washed with PBS and incubated overnight at 4 °C with specific p-H2AX (Ser139) primary antibodies followed by appropriate FITC-conjugated secondary antibody for 1 h at room temperature. A staining solution containing 0.5 μg/ml of DAPI was then applied to label DNA for 10 min at room temperature. Images were captured using an Olympus BX41 fluorescence microscope with excitation and emission filters set at 335 nm and 420 nm, respectively.

### Mitochondrial membrane potential measurement

Mitochondrial membrane potential (MMP) was assessed using a JC-10 assay kit (Abcam, ab112134). CRC cells were seeded in 24-well plates overnight, treated with the indicated drugs for 24 h, and analyzed according to the manufacturer's instructions using a BD Accuri C6 flow cytometer (BD Biosciences, San Diego, CA, USA).

### Caspase-3 activity assay

Caspase-3 activity was measured using a fluorometric caspase-3 activity assay kit according to the instructions of the manufacturer (Abcam, Germany). CRC cells were treated with 20 or 40 μM lenvatinib, with or without 80 μg/ml BP, for 48 h in 96-well plates. The caspase assay solution was added and incubated for 30 min at room temperature. Caspase-3 activity was measured using a multi-well plate reader Infinite 200 Pro Tecan^TM^ (Tecan, Mannedorf, Switzerland) with excitation and emission filters set at 400 nm and 505 nm, respectively.

### Statistical analysis

Data are presented as mean ± standard deviation (S.D.). Statistical significance was determined using the Student's t-test for values that followed a normal distribution, with * p < 0.05 versus control, ** p < 0.01 versus control, and*** p < 0.001 versus control.

## Results

### BP enhanced lenvatinib-induced cell death in CRC cells

**Figure 1:**
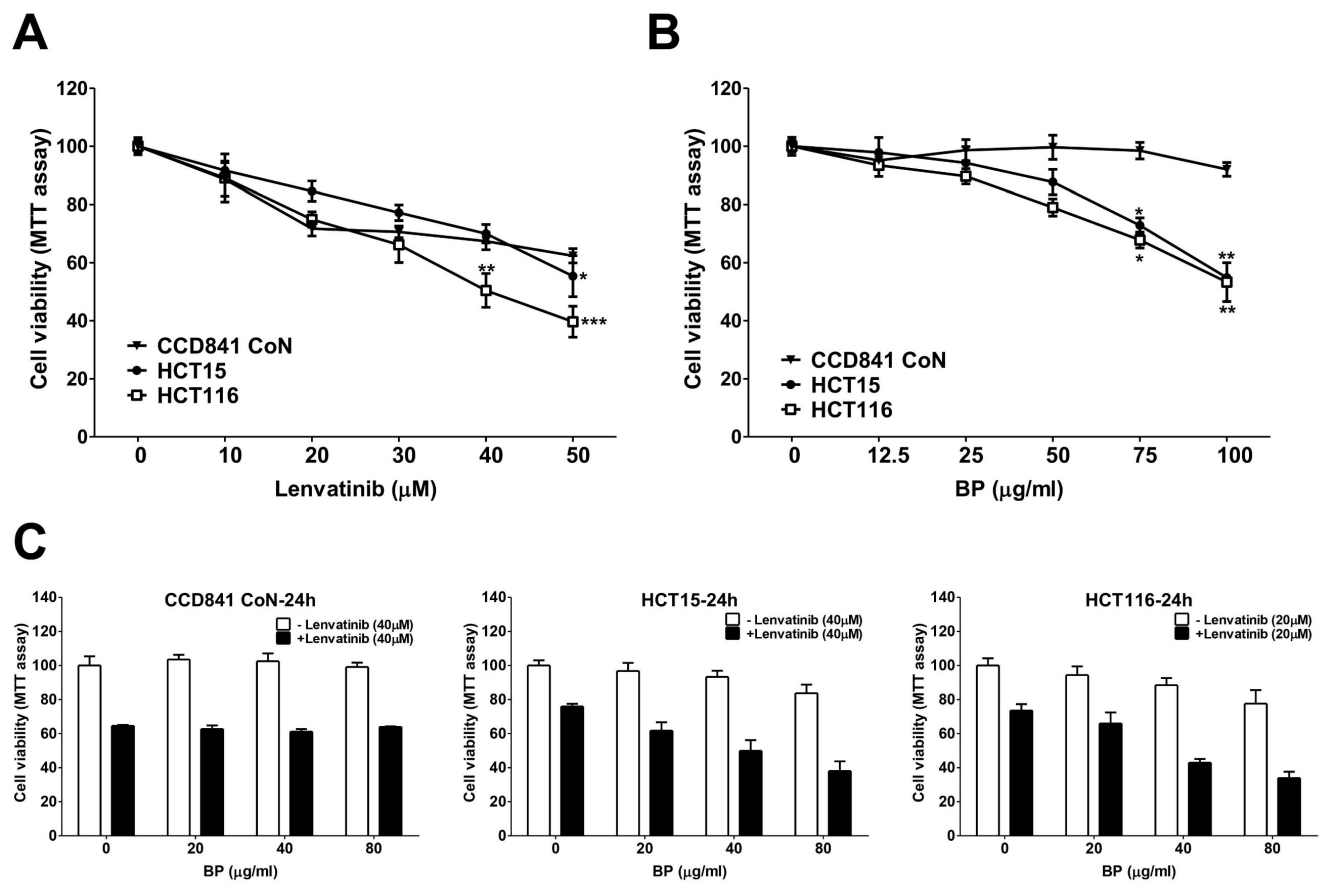
The cell viability of lenvatinib, BP, and combination treatment on CRC cell lines. CCD841 CoN, HCT15 and HCT116 cells were treated with increasing concentrations of (A) lenvatinib or (B) BP for 24 h, and cell viability was assessed using the MTT assay. (C) CCD841 CoN, HCT15 and HCT116 cells were treated with or without lenvatinib (20 or 40 μM) in the presence or absence of 20, 40 or 80 μg/ml BP for 24 h, the cell viability was assessed by MTT assay. Data are presented as mean ± S.D. obtained from three independent experiments, * p < 0.05 vs. vehicle; ** p < 0.01 vs. vehicle.

The cell viability of HCT15 and HCT116 CRC cell lines treated with lenvatinib, BP or their combination was assessed using the MTT assay (Figure 1). The IC_50_ values for lenvatinib were determined to be 57.6 μM (CCD841 CoN), 59.6 μM (HCT15) and 35.6 μM (HCT116), respectively (Figure 1A). The IC_50_ values for BP were determined to be 357.3 μg/ml (CCD841 CoN), 117.6 μg/ml (HCT15) and 110.7 μg/ml (HCT116), respectively (Figure 1B). We analyzed the potential synergistic effect of combining lenvatinib and BP on CRC cells using CompuSyn software, which computes the combination index (CI) and dose reduction index (DRI). A CI value less than 1 indicated a synergistic effect, whereas a DRI value greater than 1 demonstrated the achievable dose reduction with the synergistic combination compared to the doses of individual drugs (Table 1). The combination treatment demonstrated a dose-dependent reduction in the viability of HCT15 (20 μg/ml BP: 61.7% ± 4.9 %; 40 μg/ml BP: 49.9 % ± 6.36 %; 80 μg/ml BP: 38.0 % ± 5.7%) and HCT116 cells (20 μg/ml BP: 66.0 % ± 6.4 %; 40 μg/ml BP: 42.9 % ± 2.2 %; 80 μg/ml BP: 33.9 % ± 3.7 %) compared to lenvatinib alone (Figure 1C). However, the combination of lenvatinib (40 μM) and BP (80 μg/ml) did not exhibit a notable synergistic effect in CCD841 CoN cells, with only a 36.0 % reduction in viability observed (CI: 1.33). In contrast, the combination of lenvatinib and BP demonstrated a significant synergistic effect, with a reduction in cell viability of 61.9 % in HCT15 cells, (40 μM lenvatinib with 80 μg/ml BP, CI: 0.59) and 66.1 % in HCT116 cells (20 μM lenvatinib with 80 μg/ml BP, CI: 0.48). The combination treatment doses selected (20 or 40 μM lenvatinib and 80 μg/ml BP) were utilized in subsequent experiments with CRC cells. These results demonstrated the synergistic potential of the combination treatment for CRC cells.

### BP combined with lenvatinib promotes apoptosis and disrupts mitochondrial membrane potential in CRC cells

**Figure 2:**
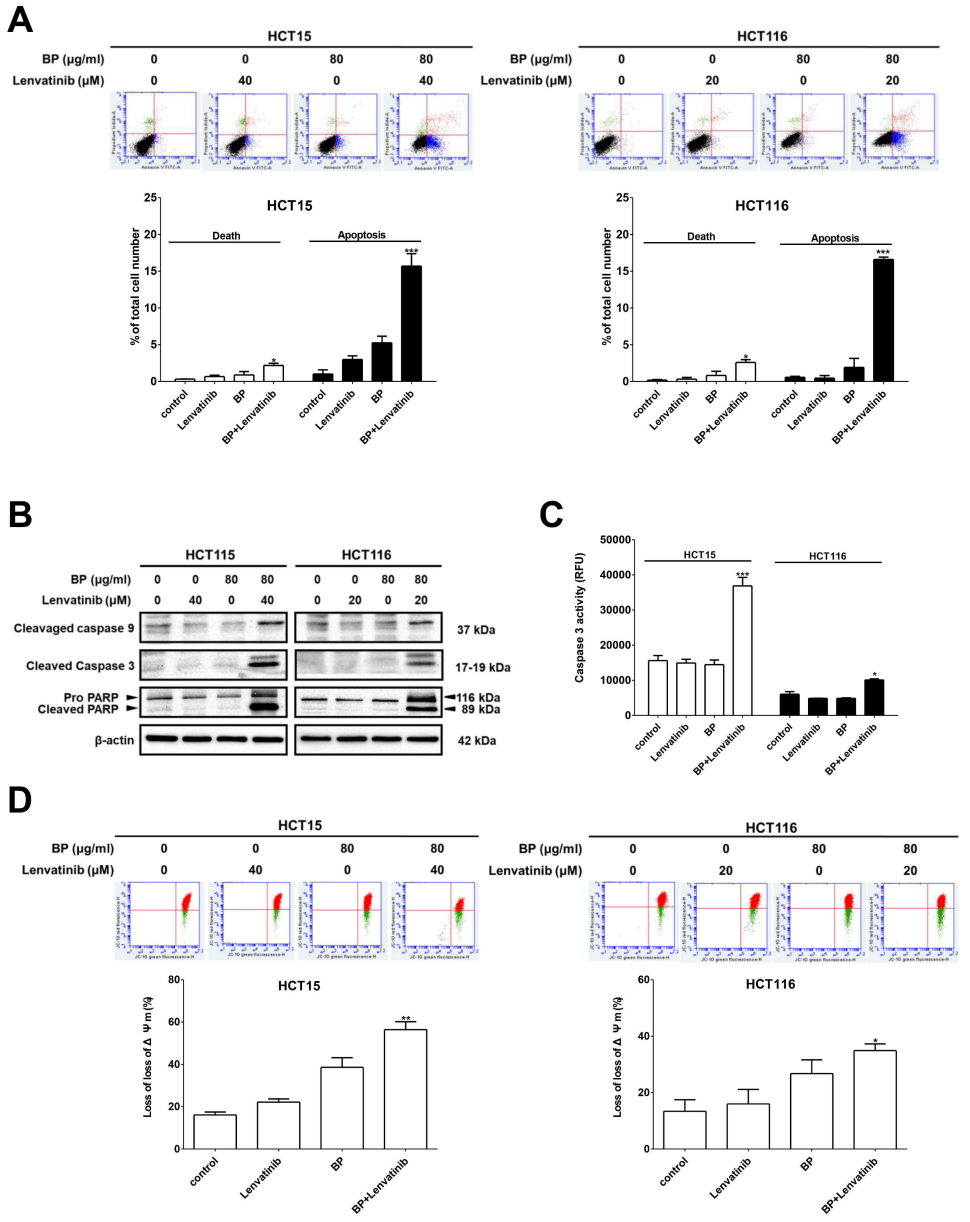
Combination treatment increased apoptosis induction in CRC cell lines. CRC cells were treated with 80 μg/ml of BP in the presence or absence of lenvatinib (20 or 40 μM) for 24 h. (A) Apoptosis induction was evaluated using annexin V-FITC staining followed by flow cytometry analysis. (B) Expression levels of cleaved caspase-9, cleaved caspase -3 and cleaved PARP were analyzed by western blot analysis. (C) Caspase-3 enzyme activity was measured using a fluorometric caspase-3 activity assay kit. (D) Mitochondria membrane potential was assessed using JC-10 staining followed by flow cytometry analysis. Data are presented as mean ± S.D. obtained from three independent experiments, * p < 0.05 vs. vehicle; ** p < 0.01 vs. vehicle.

To elucidate the underlying mechanisms by which the combination treatment induces cell death in CRC cells, apoptotic cells were identified through annexin V-FITC/PI staining and analyzed by flow cytometry. As shown in Figure 2A, the percentage of apoptotic cells significantly increased with combination treatment 15.7 ± 1.7 % (HCT15) and 16.6 ± 0.3 % (HCT116) compared to BP alone (HCT15: 5.3 ± 0.9 % and HCT116: 1.9 ± 1.2 %) or lenvatinib alone (HCT15: 3.0 ± 0.5 % and HCT116: 0.5 ± 0.4 %). Furthermore, genes involved in apoptosis induction were examined by western blot analysis. The expression levels of cleaved caspase-9, cleaved caspase-3, and cleaved PARP were dramatically upregulated after combination treatment compared to the BP or lenvatinib alone groups in CRC cells (Figure 2B). In addition, caspase-3 activity in CRC cells after combination treatment was measured using a caspase-3 activity assay. The results showed that the combination treatment significantly increased caspase-3 activity by approximately 50 % compared to the BP or lenvatinib alone groups (Figure 2C). Mitochondria play key roles in activating apoptosis and regulating cell death. Thus, we evaluated changes in the mitochondrial membrane potential (MMP, Δψm) of CRC cells treated with the combination treatment using JC-10 staining. Healthy CRC cells with intact mitochondria showed red fluorescence, while cells with depolarized mitochondria showed green fluorescence. A significant increase of green/red fluorescent ratio was detected in the combination treatment group (HCT15: 56.4 ± 3.7 %, HCT116: 34.6 ± 2.0 %) compared to the BP alone (HCT15: 34.6 ± 2.0 % and HCT116: 28.4 ± 5.2 %) or lenvatinib alone (HCT15: 38.6 ± 4.6 % and HCT116: 16.0 ± 4.2 %) groups, indicating mitochondrial damage and cellular cytotoxicity (Figure 2D). These results suggest that the combination treatment induces mitochondrial depolarization and promotes apoptosis in CRC cells.

### BP combined with lenvatinib alters cell cycle distribution in CRC cells

**Figure 3:**
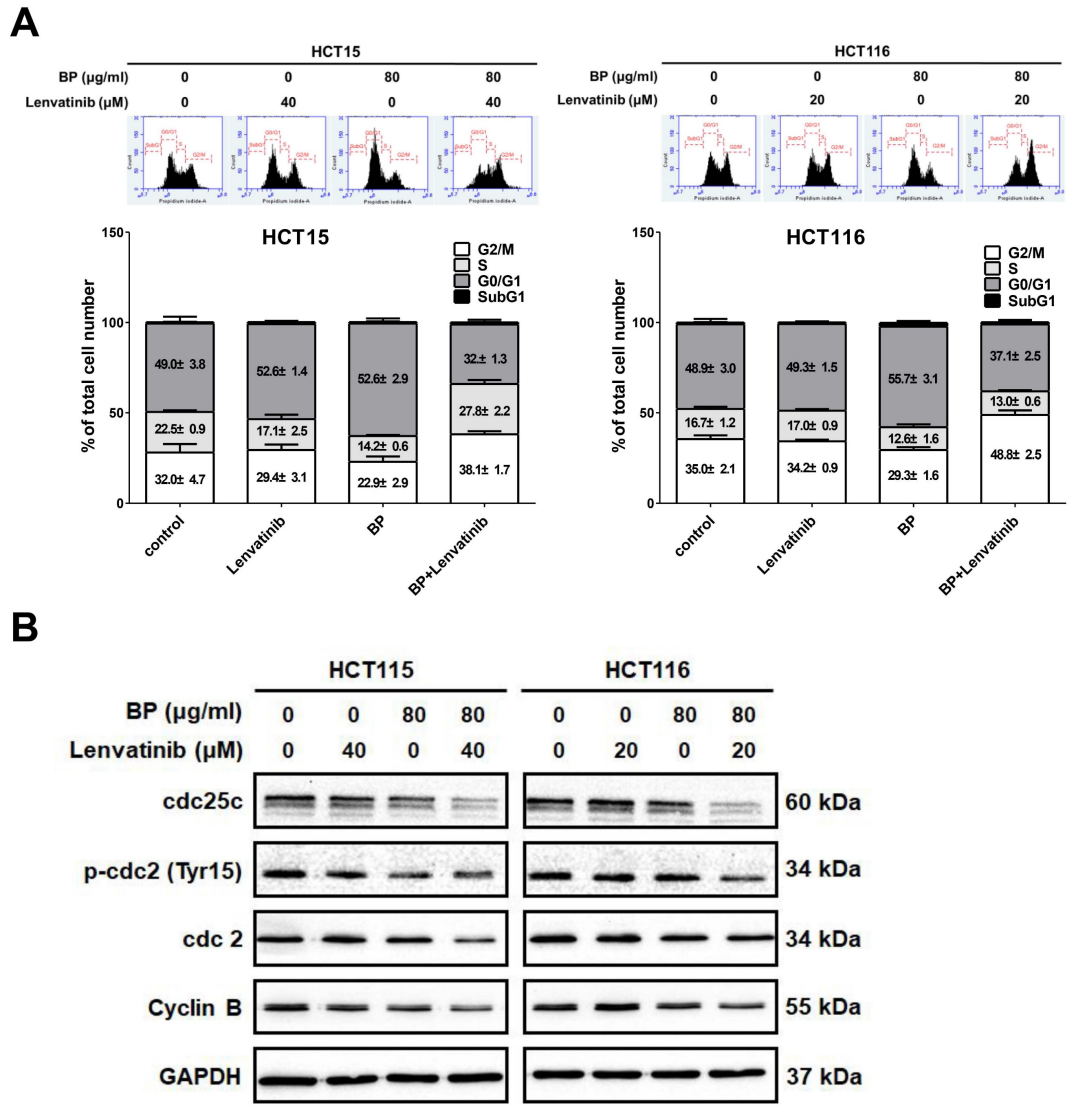
Effect of combination treatment with BP and lenvatinib on cell cycle distribution in CRC cell lines. CRC cells were treated with 80 μg/ml of BP in the presence or absence of lenvatinib (20 or 40 μM) for 24 h. (A) Cell cycle distribution was analyzed by flow cytometry. Data are presented as mean ±S.D. obtained from three independent experiments, * p < 0.05 vs. vehicle; ** p < 0.01 vs. vehicle (B) Protein expression levels of cdc25c, p-cdc2 (Tyr15), cdc2, cyclin B and GAPDH were assessed using western blot analysis.

To determine whether the proliferation inhibition induced by the combination of BP and lenvatinib is associated with alterations in cell cycle progression, we analyzed the cell cycle distribution of CRC cells using propidium iodide (PI) staining and examined the DNA content via flow cytometry. As shown in Figure 3A, the combination treatment significantly increased the proportion of cells accumulating in the G2/M phase in HCT15 (from 32.0 ± 4.7 % to 38.1 ± 1.7 %) and HCT116 cells (from 35.0 ± 2.1 % to 48.8 ± 2.5 %). To further elucidate the mechanisms underlying G2/M phase arrest, we examined the expression levels of G2/M regulatory proteins, including cyclin B, p-cdc2 (Tyr15), cdc2, and cdc25c, by western blot analysis (Figure 3B). The results showed that the combination treatment decreased the phosphorylation of p-cdc2 at the Tyr15 site, and downregulated the expression of cdc25, cdc2 and cyclin B. Together, these findings suggest that the combination of BP and lenvatinib induces G2/M cell cycle arrest by modulating G2/M phase regulatory proteins in CRC cells.

### BP combined with lenvatinib induces ROS generation and DNA damage in CRC cells

**Figure 4:**
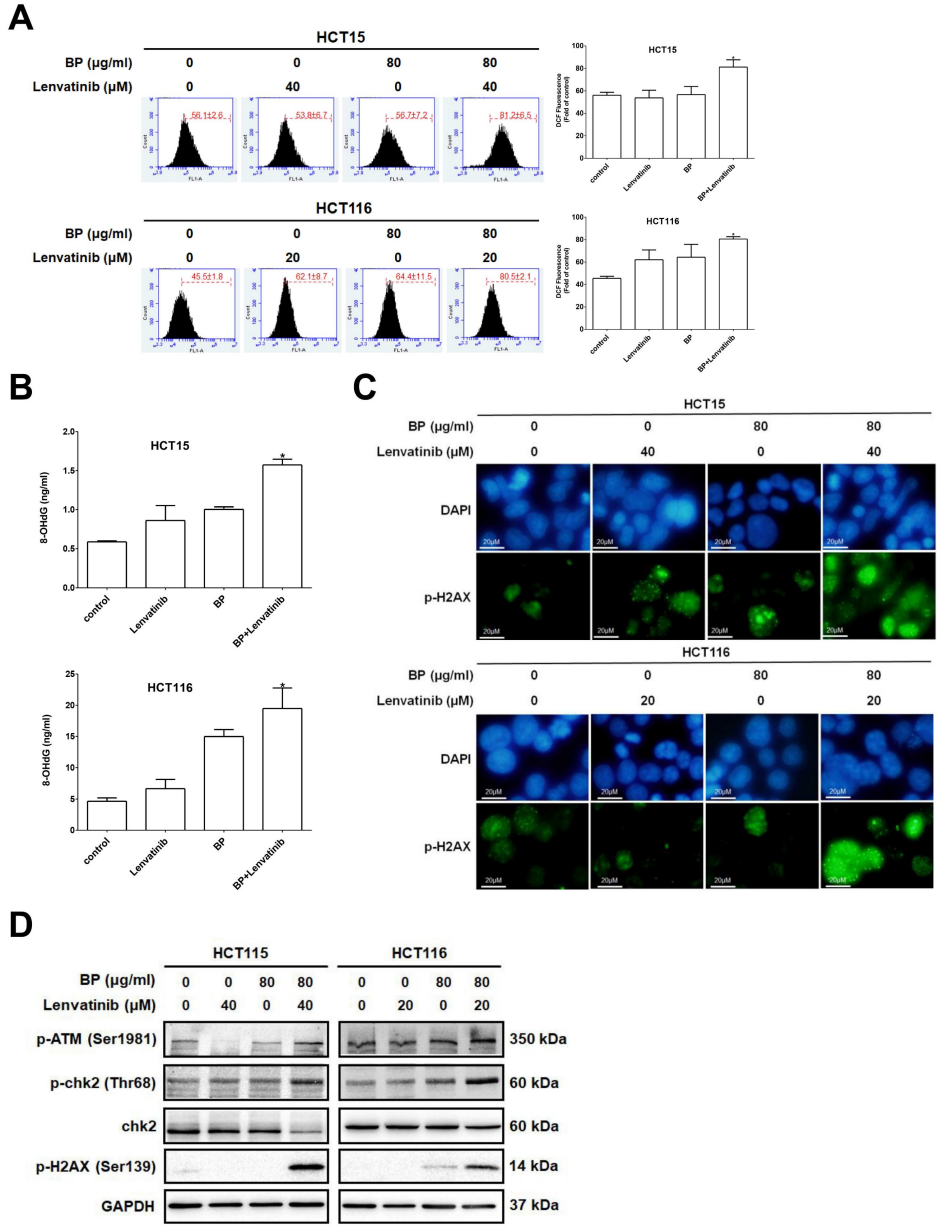
ROS-induced DNA damage was enhanced by combination treatment in CRC cell lines. CRC cells were treated with lenvatinib (20 or 40 μM) in the presence or absence of 80 μg/ml of BP for 24 h. (A) ROS generation following the indicated treatments was measured by DCFDA staining, and fluorescence intensity was analyzed by flow cytometry. (B) 8-OHdG levels following the indicated treatments were measured using an oxidative DNA damage ELISA kit and quantified with a microplate ELISA Reader at 450 nm wavelength. (C) The expression of p-H2AX after indicated treatment was assessed by immunofluorescence analysis (p-H2AX positive cells appeared green, while the cell nuclei were stained blue with DAPI). Scale bar: 20 μm. (D) The expression levels of p-ATM (Ser1981), p-chk2 (Thr68), chk2, p-H2AX (Ser139) and GAPDH after the indicated treatment was analyzed by western blot. Data are presented as mean ±S.D. obtained from three independent experiments, * p < 0.05 vs. vehicle; ** p < 0.01 vs. vehicle.

The combination treatment of BP and lenvatinib disrupted MMP, which may cause excessive ROS generation leading to cell death. To assess the level of intracellular ROS induced by BP, lenvatinib and their combination, cells were treated with the indicated treatment for 24 h and then subjected to DCFDA staining followed by flow cytometry analysis (Figure 4A). Combination treatment significantly increased intracellular ROS levels to 81.2 ± 6.5 % (HCT15) and 80.5 ± 2.1 % (HCT116) compared to BP alone (HCT15: 56.7 ± 7.2 % and HCT116: 64.4 ± 11.5 %) or lenvatinib alone (HCT15: 53.8 ± 6.7 % and HCT116: 62.1 ± 8.7 %) groups in CRC cells. To further evaluate the impact of ROS on DNA damage, we measured the levels of 8-OHdG, a well-established biomarker of oxidative DNA injury and carcinogenesis 31. Cells were treated with indicated drugs for 24 h, and 8-OHdG expression levels were measured and quantified using an oxidative DNA damage ELISA assay (Figure 4B). The results showed that the combination treatment significantly increased the 8-OHdG expression levels to 1.57± 0.07 ng/ml (HCT15) and 19.47 ± 3.29 ng/ml (HCT116) compared to BP alone (HCT15: 1.00 ± 0.03 ng/ml and HCT116: 14.98 ± 1.12 ng/ml) or lenvatinib alone (HCT15: 0.86 ± 0.19 ng/ml and HCT116: 6.63 ± 1.48 ng/ml) groups in CRC cells. To investigate whether the observed DNA damage involved double-stranded DNA breaks (DSBs), we examined the phosphorylation of histone H2AX at Ser139 (γ-H2AX), a key biomarker of DSBs and DNA repair, using immunofluorescence staining. As shown in Figure 4C, γ-H2AX foci (green fluorescence) were markedly increased after combination treatment for 24 h compared with BP or lenvatinib alone groups. In addition, ROS-induced DNA damage response triggers activation of the ATM (ataxia telangiectasia mutated)/chk2 pathway, leading to cell cycle arrest or apoptosis. To verify its activation, we further examined the protein expression profiles of the ATM/chk2 pathway by western blot analysis (Figure 4D). The results showed that the expression levels of p-ATM (Ser1981), p-chk2 (Thr68) and p-H2AX (Ser139) were dramatically increased after combination treatment for 24 h compared with BP or lenvatinib alone groups in CRC cells. Together, these findings demonstrate that the combination of BP and lenvatinib significantly increases ROS accumulation and DNA damage in CRC cells, partly through activation of the ATM/chk2 signaling pathway, thereby contributing to enhanced cytotoxicity.

## Discussion

Despite the progress in developing diagnostic tools and treatment regimens for CRC, novel therapeutic strategies that sensitize tumors to standard chemotherapy and overcome tumor resistance are urgently needed for CRC patients. In this study, the plant-derived bioactive compound BP and lenvatinib were investigated for their efficacy in combination treatments of CRC cells. We demonstrated that BP sensitized CRC cells to lenvatinib-induced growth inhibition and apoptosis induction. Moreover, the combination treatment mediated these effects by inducing ROS accumulation, resulting in mitochondrial dysfunction and DNA damage response in CRC cells. Our results also showed that the combination treatment induced cell cycle arrest at the G2/M phase via downregulation of cell cycle regulatory proteins such as cyclin B1, cdc25c, and cdc2.

Lenvatinib, a well-known multi-targeted tyrosine kinase inhibitor, has been approved by the FDA for the treatment of several cancer types, but not for CRC. Therefore, the feasibility of lenvatinib for CRC treatment is currently being explored. Several reports have demonstrated the anti-tumor activity of lenvatinib in CRC cell lines and CRC xenograft mouse models both* in vitro* and* in vivo* 8, 9. Furthermore, a phase II clinical trial indicated promising clinical efficacy of lenvatinib in CRC treatment and showed good tolerability in patients with metastatic CRC after unsuccessful standard chemotherapy 10. Another clinical trial has entered phase III and reported that the combination of lenvatinib and pembrolizumab in CRC showed promising trends in survival and response rates compared to standard treatment, although results were not statistically significant (NCT04776148). Due to the limited activity, drug resistance, and restricted efficacy of lenvatinib monotherapy, it is critical to develop lenvatinib-based combination therapies for CRC patients 32-34.

BP, a bioactive compound derived from the widely used traditional Chinese medicine (TCM) herb *Angelica sinensis* (Danggui). It exerted anti-proliferation activities against various types of cancer including lung, brain, colon, breast and liver cancers. The anticancer mechanisms are diverse and involve the induction of both mitochondrial-dependent (intrinsic) and mitochondrial-independent (extrinsic) apoptotic pathways. In glioblastoma and HCC, BP has been shown to upregulate the expression of caspase-8 and caspase-9, the initiator caspases of both apoptotic pathways, resulting in apoptosis and cell death 16, 35. Furthermore, studies have demonstrated that BP can induced intrinsic apoptotic pathway by upregulating caspase-9 and caspase-3 activation in prostate, bladder, breast and ovarian cancers 21, 30, 36-38. Previous report indicated that the phytochemicals induce mitochondrial-mediated apoptosis by affecting the mitochondrial permeability transition (MPT) pore and causing mitochondrial membrane depolarization in CRC 39. Chemotherapy agents such as 5-FU were found to induce apoptosis in CRC by causing a loss of MMP and activating caspases 40. In this study, our results showed that the combination treatment increased MMP loss and induced apoptosis by upregulating the expression levels of cleaved caspase-9, cleaved caspase -3, and cleaved PARP in CRC cells. These results suggest that the combination treatment induces mitochondrial-mediated apoptosis by regulating MPT.

Recent studies have indicated potential benefits of combining chemotherapy with Chinese herbal medicine for CRC treatment. For instance, the combination of 5-fluorouracil (5-FU) and demethylzeylasteral and cisplatin with luteolin have shown synergistic effects 41, 42. Moreover, certain plant-derived compounds, including BP, have shown safer profiles and minimal toxicity to non-cancerous cells compared to standard chemotherapy drugs such as 5-FU, oxaliplatin, capecitabine in CRC treatment 43-45. BP has also been investigated for its synergistic effects when combined with chemotherapy drugs such as cisplatin in bladder cancer and 5-FU in CRC 20, 21. In this study, our results revealed that lenvatinib decreased the proliferation of both normal colon epithelial cells (CCD841 CoN) and CRC cells (HCT15 and HCT116), whereas BP exhibited minimal toxicity to normal colon epithelial cells compared to CRC cells. Notably, the combination of lenvatinib with BP was more effective at inhibiting CRC cell growth while maintaining low toxicity to non-cancerous cells. These findings implicated that the combination treatment may offer synergistic anti-cancer effects with reduced systemic toxicity.

ROS and redox homeostasis are critical for various biological processes, but excessive ROS accumulation can induce cellular damage and lead to cell death 46, 47. Previous studies have demonstrated that numerous chemotherapeutic agents can induce increased ROS production in cancer cells 48, 49. Elevated intracellular ROS levels have been shown to inhibit cell proliferation and promote apoptosis in myeloma and CRC cells 50, 51. ROS can directly induce oxidative DNA damage and activate the DDR by forming mutagenic 8-OHdG. These adducts, along with single-strand breaks (SSBs) or DSBs induced by chemotherapy agents, contribute to DNA damage, leading to cell cycle arrest and cell death 52-54. In addition, pro-oxidant compounds such as olaparib and veliparib induce ROS generation and DNA damage, emphasizing ROS-dependent therapeutic strategies for CRC 55. Modulators targeting ROS and redox signaling pathways have been evaluated in combination with chemotherapy in clinical trials, and some have demonstrated efficacy 22. Our study revealed that the combination of lenvatinib and BP resulted in elevated levels of ROS, leading to the accumulation of DNA damage and the formation of 8-OHdG, which is a marker of oxidative DNA damage in CRC cells. Therefore, inducing excessive ROS might be an effective strategy for combating CRC.

When a DDR occurs, the ATM protein and its downstream targets are activated to induce DNA repair and transient cell cycle arrest to ensure chromosome stability. DSBs induced by radiation or cytotoxic agents initiate the formation of γ-H2AX foci, which are phosphorylated by kinases such as ATM and ATR in the PI3K pathway, facilitating DNA damage assessment, particularly in cancer treatment 56. Consistent with these findings, our results revealed that the combination treatment increased the phosphorylation of H2AX at Ser139, representing a general cellular response to processes involving DSBs in DNA. Additionally, our results also showed that the combination treatment induced ATM activation, which led to the phosphorylation of Chk2 (Thr68) and Cdc2 (Tyr15) and resulted in G2/M cell cycle block. Previous studies have indicated that Chk2, activated by ATM or ATR, played a crucial role in the cell cycle response to DNA damage 57. It regulates the inactivation of cdc25 to prevent the activation of cdc2/cyclin B, which is responsible for cell arrest at the G2/M transition 58. In breast cancer, BP induces G2/M cell cycle arrest by affecting the expression of regulatory proteins like p-cdc25c, cyclin B1, and cdc2. Notably, BP has also been shown to enhance the radiosensitivity of breast cancer cells by increasing DNA damage and downregulating the homologous recombination repair protein Rad51 30. In CRC, the antitumor effects of BP encapsulated in a polycationic liposomal polyethylenimine and polyethylene glycol complex (LPPC) were investigated in HT-29 and CT26 cells 20. The BP/LPPC induced cell cycle arrest at the G0/G1 phase through upregulation of p53 and p21 expression and downregulation of CDK4 and Cyclin D1. In this study, BP alone similarly induced G0/G1 phase arrest in HCT15 and HCT116 CRC cells. Interestingly, the combination treatment of BP with lenvatinib decreased the expression levels of cdc25c, p-cdc2, and cyclin B, resulting in G2/M phase arrest in CRC cells. These findings suggest that the combination treatment induces DNA damage and activates the ATM-Chk2 pathway by accumulating ROS.

The combination of lenvatinib and BP exerts a synergistic antitumor effect that extends beyond the simple additive effects of apoptosis and ROS-mediated cytotoxicity. Accumulating evidence suggests that the synergistic effect of this combination treatment may involve the coordinated suppression of epithelial-mesenchymal transition (EMT) and robust modulation of the DDR. Lenvatinib has been shown to inhibit pro-metastatic EMT signaling by targeting receptor tyrosine kinases and regulating the TGF-β1/Smad3 and PI3K/AKT pathways, thereby reducing the migratory and invasive capacities of cancer cells 59, 60. It has been demonstrated that BP exerts comparable effects by downregulating EMT markers such as N-cadherin and vimentin, while restoring E-cadherin expression, leading to reduced motility and invasion in various solid tumors, including CRC 20, 30. Furthermore, both agents perturb redox homeostasis and interfere with DNA repair mechanisms. Lenvatinib has been shown to promote ROS accumulation and ferroptosis through the inhibition of FGFR4 and GPX4 26. Conversely, BP has been observed to enhance oxidative stress and suppresses the homologous recombination repair protein Rad51, resulting in increased DSBs and γ-H2AX formation 22, 26, 30. These cooperative effects may induce an overload of DDR signaling via ATM-Chk2 activation, which can result in irreversible G2/M arrest and subsequent apoptotic cell death 56, 61. Collectively, these findings imply that the combination treatment of BP with lenvatinib induces a synthetic vulnerability by simultaneously promoting oxidative DNA damage and impeding EMT-driven metastatic processes, thereby providing a mechanistic rationale for their synergistic antitumor efficacy.

Resistance to lenvatinib remains a major clinical obstacle, often arising from adaptive RTK reactivation, EMT-associated phenotypic plasticity, enrichment of stem-like tumor subpopulations, and upregulation of antioxidant and DNA-repair programs that attenuate ROS-mediated cytotoxicity 27, 59. BP has been observed to exert an opposing effect on several of these adaptive mechanisms. By augmenting the levels of intracellular ROS and lipid peroxidation, BP lowers the threshold for ferroptosis and oxidative apoptosis initiated by lenvatinib-mediated FGFR4 inhibition. Simultaneously, BP suppresses Rad51-dependent homologous recombination, thereby reducing the ability of tumor cells to repair DNA damage induced by lenvatinib 30, 62. Moreover, lenvatinib's inhibition of EMT signaling complements BP's anti-EMT and anti-stemness properties, collectively restricting the emergence and propagation of resistant tumor clones 36. Through these intersecting mechanisms, the combination of BP with lenvatinib not only enhances cytotoxic potency but may also prevent or delay the onset of drug resistance by disrupting the molecular adaptations that sustain tumor survival.

BP has demonstrated robust antitumor activity in a variety of *in vivo* models, including glioblastoma multiforme (GBM), gastric cancer, and ovarian cancer, where it effectively suppresses tumor growth and induces apoptosis 35-37. Notably, BP has advanced into early clinical application via the implementation of local delivery systems, such as the *Cerebraca Wafer*. This approach has yielded durable responses and favorable safety profiles in glioblastoma patients without significant systemic toxicity 63. This clinical tolerability suggests that BP, administered at locally sustained doses equivalent to 75 mg per wafer, could be adapted to other solid tumors such as CRC. BP has been demonstrated to inhibit oncogenic signaling through the suppression of Axl and mTOR, to reduce MGMT-mediated chemoresistance, and to induce oxidative stress-driven mitochondrial dysfunction 63-65. Given the prevalence of dysregulation of Axl and mTOR in CRC, with the concomitant contribution of these pathways to EMT and therapeutic resistance, the targeting of these pathways with BP has the potential to restore chemosensitivity and suppress metastatic progression 66, 67. These existing *in vivo* data for BP, combined with the proven clinical efficacy and safety of lenvatinib 68, 69, provide a basis for the feasibility of combination therapy. However, further investigation is necessary to elucidate the safety, efficiency, and mechanism of action of this combination treatment through *in vivo* experiments.

## Conclusions

**Figure 5:**
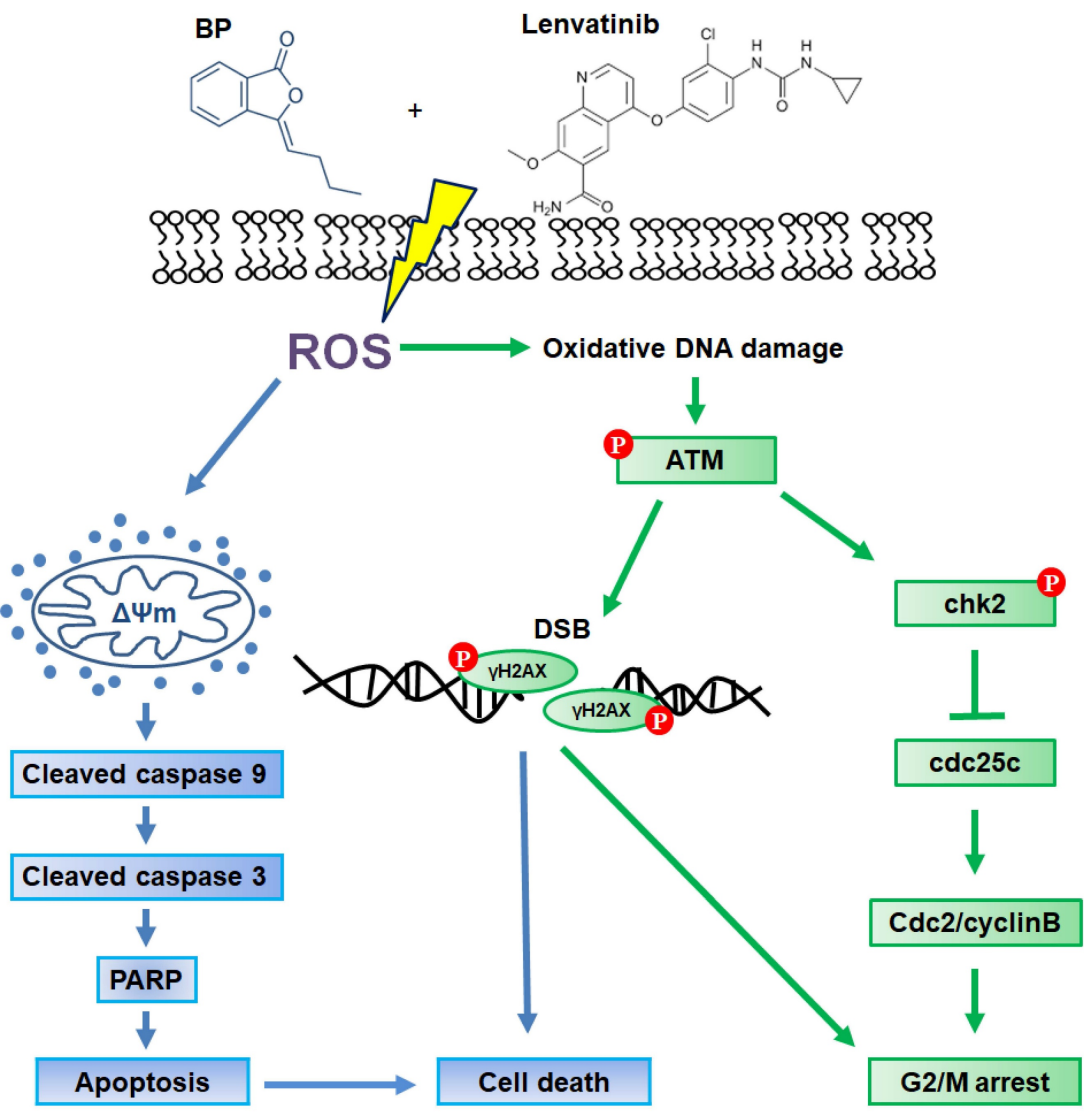
Schematic representation of the proposed mechanism underlying the anti-tumor effects of combination treatment with lenvatinib and BP treatment in CRC cells. The combination treatment enhanced cell apoptosis through loss of mitochondrial membrane potential (ΔΨm) and induced G2/M cell cycle arrest via the ATM-Chk2 signaling pathway. These effects were attributed to ROS-mediated DNA damage, leading to cell death in HCT15 and HCT116 CRC cells.

In summary, the present study demonstrates that the combination treatment of BP and lenvatinib exerts potent synergistic antitumor activity against CRC cells by inducing ROS-induced DNA damage and apoptosis. In addition, the combination treatment induces cell cycle G2/M arrest through the activation of the ATM-Chk2 signaling pathway. As illustrated in Figure 5, these findings highlight the potential of this combination treatment as a promising therapeutic strategy for CRC. Nevertheless, while the present findings provide compelling mechanistic evidence *in vitro*, further *in vivo* and translational studies are necessary to evaluate safety, efficacy, and potential resistance mechanisms before clinical application.

## Figures and Tables

**Table 1:** CI and DRI of BP combination with Lenvatinib in CRC

Concentration		CCD 841 CoN		DRI	
BP (μg/ml)	**Lenvatinib (μM)**	**fa**	**CI**	**BP**	**Lenvatinib**
20	40	0.37	1.11	17.24	0.95
40	40	0.39	1.09	9.11	1.02
80	40	0.36	1.33	4.19	0.91
Concentration		**HCT15**		**DRI**	
BP (μg/ml)	**Lenvatinib (μM)**	**fa**	**CI**	**BP**	**Lenvatinib**
20	40	0.38	0.94	9.80	1.19
40	40	0.50	0.72	7.11	1.72
80	40	0.62	0.59	5.17	2.50
Concentration		**HCT116**		**DRI**	
BP (μg/ml)	**Lenvatinib (μM)**	**fa**	**CI**	**BP**	**Lenvatinib**
20	20	0.34	0.90	7.00	1.33
40	20	0.57	0.52	8.43	2.46
80	20	0.66	0.48	6.02	3.16

**Abbreviation:** CI, combination index; DRI, dose reduction index; fa, fraction affected; BP, N-butylidenephthalide; CRC, colorectal cancer.

## Abbreviations

CRC: Colorectal Cancer
BP: N-butylidenephthalide
8-OHdG: 8-hydroxy-2-deoxyguanosine
ROS: reactive oxygen species
JC-10: 5,6-Dichloro-1,1',3,3'-tetraethyl-imidacarbocyanine iodide
FITC: fluorescein isothiocyanate
MTT: 3-(4,5-dimethyl-2-yl)-2,5-diphenyltetrazolium bromide
PBS: phosphate buffered saline
SDS-PAGE: sodium dodecyl sulfate-polyacrylamide gel electrophoresis
